# An Updated Panorama of “Living Low-Training High” Altitude/Hypoxic Methods

**DOI:** 10.3389/fspor.2020.00026

**Published:** 2020-03-31

**Authors:** Olivier Girard, Franck Brocherie, Paul S. R. Goods, Gregoire P. Millet

**Affiliations:** ^1^School of Human Sciences, Exercise and Sport Science, University of Western Australia, Perth, WA, Australia; ^2^Laboratory Sport, Expertise and Performance, EA 7370, French Institute of Sport (INSEP), Paris, France; ^3^Western Australian Institute of Sport (WAIS), Perth, WA, Australia; ^4^Faculty of Biology and Medicine, Institute of Sport Sciences, University of Lausanne, Lausanne, Switzerland

**Keywords:** live low train high, altitude training, simulated altitude, systemic hypoxia, local hypoxia

## Abstract

With minimal costs and travel constraints for athletes, the “living low-training high” (LLTH) approach is becoming an important intervention for modern sport. The popularity of the LLTH model of altitude training is also associated with the fact that it only causes a slight disturbance to athletes' usual daily routine, allowing them to maintain their regular lifestyle in their home environment. In this perspective article, we discuss the evolving boundaries of the LLTH paradigm and its practical applications for athletes. Passive modalities include intermittent hypoxic exposure at rest (IHE) and Ischemic preconditioning (IPC). Active modalities use either local [blood flow restricted (BFR) exercise] and/or systemic hypoxia [continuous low-intensity training in hypoxia (CHT), interval hypoxic training (IHT), repeated-sprint training in hypoxia (RSH), sprint interval training in hypoxia (SIH) and resistance training in hypoxia (RTH)]. A combination of hypoxic methods targeting different attributes also represents an attractive solution. In conclusion, a growing number of LLTH altitude training methods exists that include the application of systemic and local hypoxia stimuli, or a combination of both, for performance enhancement in many disciplines.

## Introduction

Individual- or team-sport athletes are constantly looking for training innovation to gain a competitive edge. Altitude/hypoxic training is often viewed as the practice adopted by athletes who live and train for several weeks at moderate natural altitude (1,800–2,500 m) in the lead up to competition, termed “living high-training high” (LHTH) (Millet et al., 2010). Since the 1968 Olympic Games, sojourns at terrestrial altitude have become popular among endurance athletes to improve their aerobic performance, with a more recent development involving performing some training nearer to sea level, also known as “living high-training low” (LHTL) (Levine and Stray-Gundersen, 1997). In recent times, altitude training has grown in popularity with team sports (Girard et al., 2013). However, traveling to a mountain region (hypobaric hypoxia) is not always feasible (i.e., travel time, athlete engagement, expenses) for all but the really top athletes or squads. Additionally, access to altitude hotel facilities (normobaric hypoxia) for permanent residence at simulated altitude is costly and logistically difficult for large numbers of athletes.

**Figure 1:**
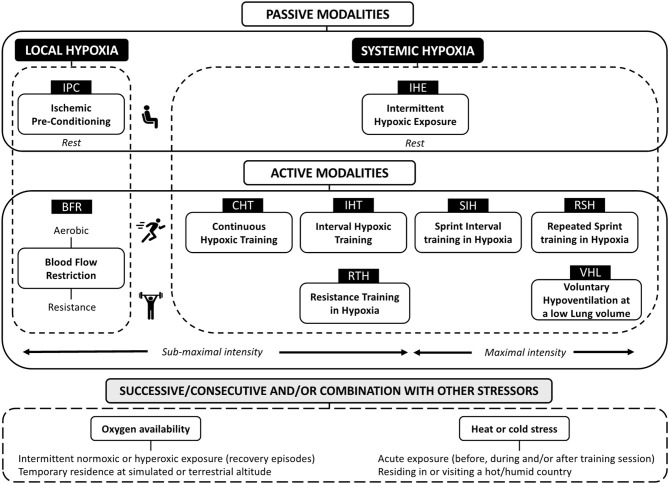
Schematic overview of “living low-training high” (LLTH) altitude/hypoxic methods.

Vision of athletes gasping for breath when undertaking training programs in oxygen (O_2_) controlled rooms is increasingly common. With minimal costs and travel constraints for athletes, the “living low-training high” (LLTH) approach is becoming an important intervention for modern sport. Athletes implementing LLTH methods receive discrete and relatively brief intervals of exposure to hypoxia that typically last <2 h, at rest or while training, 2–5 times per week. The popularity of the LLTH model of altitude training is also associated with the fact that it only causes a slight disturbance to athletes' usual daily routine, allowing them to maintain their regular lifestyle in their home environment (McLean et al., 2014). Moreover, sleep quality or recovery is well preserved since the athletes do not sleep in hypoxia. The anticipated benefits of LLTH may vary widely across individuals, in particular when the focus of their training also differs (preferential aerobic *vs*. anaerobic adaptations) (Figure 1).

Nonetheless, it is worth mentioning that the LLTH model is not new. In fact, its effects on the human body have been studied as early as the 1940s by Russian researchers. Dating back to World War II, the former Soviet Union used hypobaric chambers to condition their pilots to fly open cockpit airplanes at altitudes higher than 7,000 m (Sirotinin, 1940). The technological development of new tools that either decrease the atmospheric pressure in the room (e.g., hypobaric chambers) or reduce the fraction of O_2_ in the inspired air (FiO_2_) by diluting it with extra nitrogen or filtering out O_2_ (e.g., altitude tents, hypoxicator machines) has prompted renewed interest in LLTH interventions (Wilber, 2007). In this perspective article, we first discuss the evolving boundaries of the LLTH paradigm and its practical applications for athletes by providing an updated panorama of available methods (Figure 1) and finally we highlight areas for future research.

## Passive Modalities

Research into the effects of hypoxia under passive conditions has involved exposing individuals intermittently to systemic [intermittent hypoxic exposure at rest (IHE)] or local [(ischemic preconditioning (IPC)] hypoxia while seated at rest.

### Intermittent Hypoxic Exposure at Rest (IHE)

IHE refers to the use of “brief” periods (3–6 min) of relatively severe levels of hypoxia (FiO_2_ = 0.15–0.09; ~2,800–5,500 m simulated altitude) interspersed with normoxic episodes of similar duration. Intermittent applications of “chronic” hypoxic exposures lasting 30 min to 5 h have also been introduced for high altitude expeditions pre-acclimatization protocols, with the main purpose of improving well-being (i.e., reducing the acclimatization time) and performance at altitude (Serebrovskaya, 2002). A meta-analysis indicated that IHE may improve performance in sub-elite, but not elite, performers (Bonetti and Hopkins, 2009). However, the effect observed on performance and selected physiological variables is contentious, and this strategy may not improve endurance capacity any more than regular training (Bärtsch et al., 2008). Moreover, an IOC expert group pointed that the underlying mechanisms of IHE remain obscure and concluded that “*the use of intermittent hypoxic exposure does not increase sea-level performance and is not recommend. Further research in this area with respect to improving endurance performance does not seem warranted”* (Lundby et al., 2012).

### Ischemic Preconditioning (IPC)

IPC is a procedure causing tissue ischemia via circumferential compression of limb(s) followed by reperfusion in a repeated, cyclic manner (Murry et al., 1986). Common IPC protocols include three or four cycles of 5 min circulatory occlusion and reperfusion that are usually completed 30–45 min prior to a given exercise. Despite large between-study variability, IPC benefits include a subsequent 1–5% gain in time-trial performance and aerobic capacity (Salvador et al., 2016). IPC is also effective for improving performance at altitude (Paradis-Deschênes et al., 2018). While it represents an attractive ergogenic aid, likely related to local hyperaemia inducing accelerated/greater tissue re-/oxygenation dynamics, mechanisms remain poorly resolved (Incognito et al., 2016). Additionally, rather than a one-time intervention administered in acute settings, another novel application of IPC is to use it chronically to evoke favorable vascular (i.e., larger brachial artery flow-mediated dilatation; Jones et al., 2015) and molecular (i.e., genes involved in mitochondrial oxidative metabolism; Depre et al., 2010) adaptations.

## Active Modalities

Possible improvements in exercise capacity resulting from LLTH interventions depend on the type and intensity of the workout considered—this includes using either local [blood flow restricted (BFR) exercise] and/or systemic hypoxia [continuous low-intensity training in hypoxia (CHT), interval hypoxic training (IHT), repeated-sprint training in hypoxia (RSH), sprint interval training in hypoxia (SIH) and resistance training in hypoxia (RTH)].

### Local Hypoxia

#### Blood Flow Restriction

BFR exercise requires the continuous or cyclical application of an inflatable cuff or elastic wraps around a limb. Limiting the blood supply to and return from the contracting muscle (i.e., proximal to the muscles to be trained) produces a local hypoxic stress (Wernbom et al., 2008). In well-trained athletes this practice can induce hypertrophy and strength gains (albeit small) during low-load dynamic exercise training, while it may not cause noticeable modifications under normal circumstances (Scott et al., 2016). Training with BFR enhances performance and K^+^ regulation during intense exercise, in turn improving blood flow to exercising muscles and muscle anti-oxidant function (Christiansen et al., 2019). Although the magnitude of gains varies between modalities, this effect is true for both low-load resistance (e.g., weight lifting at 20–30% of 1 repetition maximum) and aerobic (e.g., cycling at 50–70% of maximal heart rate) BFR exercises. Despite BFR exercise typically utilizing lower training loads than conventional exercise (i.e., therapeutic exercise loading for return to performance athletes; Korakakis et al., 2018), repeated “all-out” efforts can also be performed with application of moderate cuff pressures (i.e., 40–60% of maximal occlusion pressure) to restrict venous return while preserving arterial inflow (Willis et al., 2019). For evidence-based recommendations when implementing BFR exercise, the reader is referred to Patterson et al. (2019).

### Systemic Hypoxia

#### Submaximal Exercise

Continuous hypoxic training (CHT) corresponds to continuous sub-maximal training sessions in hypoxia, usually in order to improve endurance-based performance (McLean et al., 2014). Similarly to IHE, IHT refers to using moderate hypoxic exposures (i.e., generally 2,500–3,500 m although more severe elevations up to 5,500 m can also be used; Sanchez and Borrani, 2018), but during high-intensity exercise (>70% of maximal heart rate) interspersed with similar or shorter duration recoveries, and generally performed to improve high-intensity activities (McLean et al., 2014). To date, however, the effects of CHT and IHT to further improve performance compared to sea level equivalents remain elusive (Faiss et al., 2013).

RTH involves resistance training in hypoxia in order to boost muscular strength and power production (Scott et al., 2015). Due to vastly different methodologies for implementing RTH (e.g., training programs, hypoxia severity, and participants background), improvements are not always greater than resistance training in normoxia (Ramos-Campo et al., 2018). Common features of IHT and RTH efficacy are the relatively brief inter-set recovery periods in order to provide a potent metabolic stimulus to enhance anabolic responses, beyond which is possible with matched normoxic training (Scott et al., 2015). If absolute exercise intensities are not carefully matched between hypoxic and normoxic conditions, however, determining the true effectiveness of LLTH interventions (i.e., submaximal exercise intensities) remains difficult.

#### Maximal Exercise

RSH is a recent training method that was introduced given some of the inherent limitations of IHT (e.g., lower hypoxia-induced training stimulus) (Faiss et al., 2013). Briefly, RSH is the repetition of short (<30 s) “all-out” sprints with incomplete recoveries (<60 s) in hypoxia, also including exercise-to-rest ratios typically lower than repeated-sprint ability tests/in-match scenarios to increase metabolic strain (Millet et al., 2019). The general consensus is that RSH leads to superior (1–5%) repeated-sprint ability (i.e., faster mean sprint times or higher power outputs often accompanied by a better fatigue resistance) in normoxic conditions (Brocherie et al., 2017).

When hypoxia is induced by voluntary hypoventilation at a low lung volume (VHL), which causes relatively similar hypoxemia than altitude exposure (Woorons et al., 2007), encouraging results have been reported. In highly trained rugby players, for instance, RSH-VHL has shown larger improvements in repeated-sprint performance (~60% more sprints on average during an exhaustive “open loop” test) than with an unrestricted breathing pattern (Fornasier-Santos et al., 2018). Using this technique, swimmers can also train in hypoxia while residing near sea level and expect an improved repeated-sprint ability through an accentuation of the glycolytic stimulus of their training (Trincat et al., 2017).

Sprint interval training typically involves 20-s to 30-s “all out” efforts interspersed by 3 to 5 min of passive or low-intensity active recovery episodes. This training is often presented as a time-efficient strategy to increase cardiac output, maximal O_2_ uptake, and skeletal muscle mitochondrial content to a similar or even greater extent than traditional, high-volume endurance training (Gibala and Hawley, 2017). Reduced O_2_ delivery to the muscles with hypoxic exposure increases the stress on glycolytic flux, up-regulating this energy pathway (Puype et al., 2013). Consequently, SIH is a useful exercise stimulus to maximize exercise performance both at sea level and at altitude (Kon et al., 2015).

## Combination of Various Methods

A combination of hypoxic methods targeting different attributes also represents a sounded approach (Millet et al., 2010). When implementing a “living high-training low and high” (LHTLH) intervention, for instance, athletes live high and train low except for few intense sessions in hypoxia. Reportedly, LHTL and RSH when combined can elicit concurrent aerobic and anaerobic adaptations, as manifested by a better repeated-sprint ability, in team-sport athletes (Brocherie et al., 2015). Intermittent hypoxic interval training (IHIT) is a method where hypoxia and normoxia are alternated during the same session (Millet et al., 2010). Hypoxic preconditioning (HPC), utilizing continuous or intermittent exposure to O_2_-deprived conditions, can also be superimposed to BFR to eventually augment the magnitude of the hemodynamic responses during realization of the subsequent exercise (Aebi et al., 2019). The combination of systemic hypoxia with vascular occlusion represents a promising mixture of hypoxic methods, for instance, BFR resistance exercise + RTH (Girard et al., 2019) or BFR aerobic exercise + RSH (Peyrard et al., 2019), for maximizing the training stimulus. Many training centers in altitude [Font-Romeu (France) or Colorado Springs (USA) at 1850 m combined with RTH and/or RSH in a climatic chamber] or at sea level [Aspetar hospital (Qatar), Western Australia Institute of Sport (Australia)] offer the possibility for such combination of hypoxic methods.

Although this is beyond the scope of this perspective article, there are various options for applying an hypoxic stimuli together with other environmental stressors—i.e., hot/humid air (heat stress; Takeno et al., 2001) or O_2_-enriched air (hyperoxia; Brinkmann et al., 2018)—otherwise known to independently improve physical performance (Millet et al., 2010; Gibson et al., 2019). In doing so, however, it is important to verify that absolute training quality is not blunted due to exacerbated fatigue development. For optimal implementation, the physiological stress associated with a combination of various methods or stressors can be mitigated by lower relative workload (McLean et al., 2014). Practically, this would require adjustment of duration/intensity of exercise and recovery intervals, exercise-to-rest ratio, inter-set recovery duration and/or session frequency. Whether the repetitive use of various stressors during training periods, in reference to each one alone, may also lead to further performance and physiological capacity enhancements is largely undetermined.

## On the Non-Haematological Mechanisms Underlying Adaptations

With LLTH, it is doubtful that the hypoxic dose *per se* is sufficient to induce beneficial hematological changes in red blood cell number or total hemoglobin mass (Humberstone-Gough et al., 2013). With hypoxia exposure, the transcriptional signaling and subsequent adaptations are determined at the molecular level by HIF-1α related processes (Semenza, 2000). The many common features in the signaling initiated by exercise and hypoxia suggest that the two stressors may exert a more robust stimulus. LLTH up-regulates the activity of the transcriptional factor HIF-1α (Vogt et al., 2001; Brocherie et al., 2018) that not only govern the regulatory genes for erythropoiesis but also vasodilatory responses, glycolysis and pH regulation. Reportedly, short and intense workouts characterizing LLTH may cause larger skeletal muscle tissue adaptations through the O_2_ sensing pathway [capillary-to-fiber ratio, fiber cross-section area, myoglobin content and oxidative enzyme activity] than normoxic conditions (Brocherie et al., 2018). Practically, greater buffering capacity, lactic acid tolerance and/or O_2_ extraction in the working muscle would ultimately improve exercise tolerance.

## Limitations, Challenges and Opportunities of LLTH Interventions

Despite mounting evidence of LLTH safety and efficacy, some uncertainty remains:

- While more strictly controlled and mechanistic studies are required, no single form of LLTH exists that is more effectively improving physical performance. Considering the large variability in between-subject LLTH responsiveness and the rather modest improvements in exercise capacity associated with these methods, future studies should increase their sample sizes (McLean et al., 2014). Determination of whether clamping of SpO_2_ would induce a more consistent hypoxic stimulus, eventually reducing variability of the LLTH responses across individuals (Hamlin et al., 2010), is required.
- Beyond the LLTH method considered, selection of an appropriate “hypoxic dose” (i.e., hypoxia severity, duration of a single exposure, and the number of aggregate exposures) and effective management of the training load likely influence attainment of peak physical performance (Chapman et al., 2014). Hypoxia is a stressor that may do more harm than good if it is too intense. In other words, it cannot be assumed that “more is better” regarding the LLTH dosage (Goods et al., 2014). Consequently, if the total training load (i.e., exercise-to-rest ratio, recovery times) is not adjusted accordingly, the amplified training impulse can swiftly lead to overreaching or overtraining.
- Identifying the optimal period of a competitive season to implement each LLTH method and the decay of adaptations during several days or weeks post-intervention deserve future attention. It also remains to be determined whether each LLTH method is best used as a strategy for pre-acclimatization, improved training responsiveness following an altitude training camp, accelerating musculoskeletal rehabilitation and/or preventing detraining during immobilization in injured athletes. The value of LLTH as a “performance recovery” strategy, for example, to limit the extent of muscle damage (Arriel et al., 2018), is promising.
- Aside from hypobaric chambers, normobaric reduced O_2_ rooms or terrestrial altitude, recent technological boosts open doors for military and clinical applications of LLTH methods with the widespread development of portable mask-system hypoxicator machines (Lopata and Serebrovskaya, 2012). Mobile inflatable simulated hypoxic equipment now allows swimmers or team-sport players to train in hypoxia, for example by performing swim intervals, repeated running sprints or small-side games directly in their naturally-occurring environments (Girard et al., 2013).
- Direct comparisons of the effects of VHL with other LLTH methods are required. In doing, there is also a need to better understand mechanisms at play and the potential for combination with other LLTH modalities.
- In many populations, musculoskeletal disorders such as knee osteoarthritis (e.g., older adults and/or obese individuals) or painful locomotion (e.g., injured populations awaiting surgical procedures) may outweigh possible benefits of physical activity or lead to temporary loss of fitness due to reduced mobility. The combination of slower walking/running speeds (i.e., decreased external load) and hypoxia exposure (i.e., increased internal load) is a novel intervention that in theory is metabolically similar to exercising near sea level (normoxia), yet it is associated with a reduced exercise-induced pain (Girard et al., 2017). Clinical validation of hypoxic conditioning as an innovative LLTH strategy improving therapeutic outcome beyond what is obtained today is necessary.

## Conclusion

A growing number of LLTH altitude training methods exists that include the application of systemic and local hypoxia stimuli, or a combination of both, for performance enhancement in many disciplines (Figure 1). Moving forward, elite athletes and their support team will continue to strive for refinement of best practice and the individualization of the altitude dose to ultimately reduce inter-individual variability. Translation of these innovative LLTH interventions to the clinical field is also promising.

## Author Contributions

OG drafted the manuscript and prepared Figure 1. All authors listed have edited, critically revised, and approved the final version for submission.

### Conflict of Interest

The authors declare that the research was conducted in the absence of any commercial or financial relationships that could be construed as a potential conflict of interest.
